# Development and Validation of the Primary Care Needs Assessment (PCNA) Questionnaire: A Participatory Multidimensional Approach to Identifying Health Needs

**DOI:** 10.3390/healthcare14101302

**Published:** 2026-05-11

**Authors:** Eleni Papakosta-Gaki, Anastasia Zissi, Andreas Tsounis, Evangelos Kyritsakas, Stella Ploukou, Pavlos Sarafis, Alexis Benos, Emmanouil Smyrnakis

**Affiliations:** 1 Laboratory of Primary Health Care, General Medicine and Health Research Services, Medical School, Aristotle University of Thessaloniki, 54124 Thessaloniki, Greece; papakostae@auth.gr (E.P.-G.); sploukou@auth.gr (S.P.); benos@auth.gr (A.B.); smyrnak@auth.gr (E.S.); 2 Sociology Department, University of Aegean, 81100 Mytilene, Greece; a.zissi@soc.aegean.gr; 3 Psychology Department, Aristotle University of Thessaloniki, 54124 Thessaloniki, Greece; atsounis@psy.auth.gr; 4 Social Inclusion and Education Department, National Organization for the Prevention and Treatment of Addictions (E.O.P.A.E.), 41500 Larissa, Greece; ekyritsakas@ecd.uoa.gr; 5 Department of Early Childhood Education, National and Kapodistrian University of Athens, 10680 Athens, Greece; 6 Department of Nursing, University of Thessaly, 41500 Larissa, Greece

**Keywords:** community-based participatory research, factor analysis, statistical, health services needs and demand, primary health care, psychometrics, surveys and questionnaires, validation study

## Abstract

**Highlights:**

**What are the main findings?**

- The PCNA, developed through a participatory mixed-methods design, demonstrated a stable 9-factor structure with satisfactory validity and internal consistency.
- Unmet needs were identified in mental health, access to specialized care, and structural barriers, confirming the multidimensional nature of health needs in PHC.

**What are the implications of the main findings?**

- The PCNA offers a validated, community-oriented tool to support evidence-informed planning and resource allocation in primary health care.
- Incorporating community perspectives into measurement supports a shift toward more responsive, person-centered, and context-sensitive PHC systems.

**Abstract:**

**Background/Objectives**: Assessing population healthcare needs is essential for effective primary health care (PHC) planning; however, existing approaches often rely on biomedical or service-centered frameworks (i.e., approaches focusing on utilization patterns, clinical indicators, and provider performance metrics) that may not adequately capture the multidimensional and context-dependent nature of health needs. This study aimed to develop and validate the Primary Care Needs Assessment (PCNA) questionnaire, a participatory, multidimensional, and context-sensitive instrument for assessing perceived unmet healthcare needs and contextual determinants of health in community-based PHC settings. **Methods**: A sequential mixed-methods design was employed. In the qualitative phase, focus groups with PHC professionals and semi-structured interviews with community members informed item generation. The resulting questionnaire was administered to a sample of 817 participants recruited from community and primary care settings. Exploratory factor analysis (EFA) was conducted on a subsample (*n* = 520), followed by confirmatory factor analysis (CFA) on an independent subsample (*n* = 297). Internal consistency was assessed using Cronbach’s alpha. **Results**: EFA identified a 10-factor structure explaining 55.05% of the variance. CFA supported a refined 9-factor model with good fit indices (χ^2^/df = 1.675, RMSEA = 0.048, CFI = 0.92, TLI = 0.90, SRMR = 0.06). The instrument demonstrated satisfactory internal consistency (α = 0.76). Findings also highlighted unmet needs related to mental health, access to specialized services, and structural barriers (e.g., geographic distance, financial cost, limited service availability, and organizational constraints in accessing care), underscoring the multidimensional nature of health needs. **Conclusions**: The PCNA is a valid and reliable instrument that captures the complex interplay of individual, social, and contextual factors shaping health needs in PHC. By integrating community perspectives with psychometric validation, it provides a practical tool for supporting evidence-informed planning and more responsive, person-centered PHC systems (i.e., systems that adapt service provision to community-identified priorities and evolving population needs).

## 1. Introduction

Primary health care (PHC) systems play a central role in promoting population health, preventing disease, and addressing inequalities in access to health services. As the first point of contact between communities and health systems, PHC requires planning processes that are responsive to the actual needs of the populations served. Health needs assessment (HNA) is widely recognized as a key tool for aligning health policy, service planning, and resource allocation with population needs [1,2]. By identifying gaps between existing services and population needs, HNA can support the development of more responsive and equitable health systems, reflecting persistent gaps between population needs and actual service delivery [3,4].

However, traditional approaches to health needs assessment have largely relied on epidemiological indicators, administrative data, and service utilization patterns. While these sources provide valuable information about population health status, they often fail to capture the subjective, social, and contextual dimensions of health needs experienced by individuals and communities, including needs that remain unexpressed or insufficiently recognized within health systems [5,6,7,8]. Similar limitations have also been highlighted in applied public health contexts (i.e., real-world public health practice settings, including primary care delivery, community health planning, and resource allocation processes), where needs assessment processes remain fragmented and insufficiently integrated into planning and decision-making [9,10].

Health-related needs are not limited to clinically defined conditions but also encompass perceived, expressed, and unmet needs shaped by social context and individual experience, which may not be adequately captured through service utilization or epidemiological indicators [2,9]. This is further supported by recent evidence demonstrating the persistence of unmet health needs even within systems with established service provision, including well-resourced healthcare systems [10]. In this study, we adopt a multidimensional concept of health-care need that builds on classic definitions of health needs assessment and the Andersen Behavioral Model of Health Services Use. Health-care needs are understood as needs that can benefit from health-care actions (prevention, diagnosis, treatment, rehabilitation and support), rather than only as clinically defined conditions, and include unmet needs that remain insufficiently recognized within health systems. Within Andersen’s model, need is treated as both perceived need—how people view and experience their own health status, symptoms and everyday functioning—and evaluated need, referring to more professional or objective assessments of health status and need for care [11,12,13,14,15]. Building on these perspectives and on recent work in community health-needs assessment, we distinguish three interrelated dimensions of health-care needs in primary care settings: (a) subjective health-care needs, referring to individuals’ perceived current and future health problems, functional limitations and unmet needs for care; (b) social health-care needs, arising from social and economic conditions, roles and support networks that shape both health and the capacity to seek and use care; and (c) contextual health-care needs, related to characteristics of local services and broader environments that create or amplify gaps between population needs and available PHC provision.

These three dimensions guided item generation and are reflected in the factorial structure of the Primary Care Needs Assessment (PCNA) questionnaire, which comprises factors capturing perceived and unmet clinical and functional needs (subjective dimension), social and economic strain and support deficits (social dimension), and barriers related to the organization, accessibility and responsiveness of local primary-care services (contextual dimension) [1,7,9,16,17,18,19,20,21].

This broader conceptualization underscores the need for approaches that move beyond predominantly biomedical and static assessments toward more integrative and context-sensitive models of needs assessment. In this direction, the integration of quantitative and qualitative data, as well as the inclusion of community perspectives alongside those of health professionals, has been increasingly recognized as essential for addressing the complexity of health needs in PHC settings [22,23,24,25].

Participatory approaches, particularly those grounded in community-based participatory research (CBPR), emphasize the active involvement of stakeholders in identifying priorities and shaping health interventions. Engaging community members and frontline professionals in the development of assessment tools can enhance their relevance, acceptability, and practical applicability [25,26,27], while recent applications suggest their potential contribution to capturing context-specific and community-defined health needs [20,24].

However, such approaches are often applied as context-specific tools for project design and are iteratively refined based on stakeholder feedback, without being systematically translated into standardized, psychometrically validated instruments [16,19].

A complex interplay of individual, social, and structural factors shapes health needs and health service utilization. The Andersen Behavioral Model of Health Services Use provides a widely recognized framework for understanding this complexity, distinguishing between predisposing, enabling, and need-related factors at both individual and contextual levels [11,12,13,14,15]. Within this framework, health needs are understood not solely as clinical conditions but as outcomes of dynamic interactions between perceived needs, access to services, and broader social determinants.

Several quantitative instruments have been developed to assess health needs or related constructs, including generic health status measures such as the Nottingham Health Profile (NHP) [28,29], PHC assessment tools [30,31], and condition-specific needs questionnaires [17,19]. More recently, quantitative approaches to measuring unmet healthcare needs have been implemented in population-based surveys and toolkits, using standardized questions and indicators to capture both expressed and unexpressed needs across different conditions and settings [10,18].

While these approaches provide valuable population-level insights, they often rely on predefined indicators (e.g., consultation rates, referral patterns, screening uptake) and may therefore fail to capture the contextual, subjective, and community-defined dimensions of health needs, particularly in PHC settings characterized by complex and evolving needs (e.g., workforce constraints, patient–provider relational dynamics, varying levels of community trust) [2,8,18].

However, many existing quantitative tools are either condition-specific, primarily focused on service performance, or embedded in large-scale survey frameworks, which may limit their applicability as flexible, community-oriented instruments for assessing perceived, unmet, and contextual health needs in PHC settings [1,18,21].

In addition, many existing instruments are based on predefined and predominantly biomedical categories of need, which may constrain their ability to capture the dynamic and context-dependent nature of health needs [5,8]. Widely used PHC assessment tools, such as the Primary Care Assessment Tool (PCAT) [30,31] and the Patient-Centred Primary Care Measure (PCPCM) [32,33], have contributed significantly to the evaluation of PHC performance; however, they primarily focus on service attributes and care experiences (i.e., individual encounters with health services, such as satisfaction and communication quality) rather than on the assessment of community-perceived and context-dependent health needs (i.e., broader social, environmental, and systemic factors that shape access to and utilization of care beyond the clinical encounter) [34]. Moreover, existing instruments vary considerably in their scope and psychometric robustness, with only a limited number comprehensively capturing core primary care attributes while demonstrating adequate validity and reliability [35,36]. Recent studies highlight the need for more integrative and context-sensitive approaches to needs assessment in PHC [20,36,37,38].

Taken together, these limitations highlight a gap in the availability of validated, community-oriented instruments for assessing health needs in PHC settings [1,21]. Although the Andersen Behavioral Model has been widely applied in health services research [13,14,15], its multidimensional structure is not always fully operationalized in existing assessment tools, particularly in ways that reflect the interplay of individual, social, and contextual determinants of health needs.

To address these gaps, the present study introduces the Primary Care Needs Assessment (PCNA), a multidimensional instrument designed to assess perceived, unmet, and contextual health needs within community populations in PHC settings. The instrument was developed through a mixed-methods approach integrating perspectives from PHC professionals and community members into the instrument development process.

The aim of the present study was to develop and validate a context-sensitive PCNA questionnaire and to examine its factorial structure and reliability using exploratory and confirmatory factor analysis. By integrating community perspectives, defined as the lived experiences, priorities, and self-identified needs of community members and local stakeholders, into the development and validation of a needs assessment instrument, the study endeavours to provide a structured tool for assessing population healthcare needs and to support evidence-informed planning and decision-making in PHC systems.

## 2. Materials and Methods

### 2.1. Generating the PCNA Questionnaire

**Figure 1:**
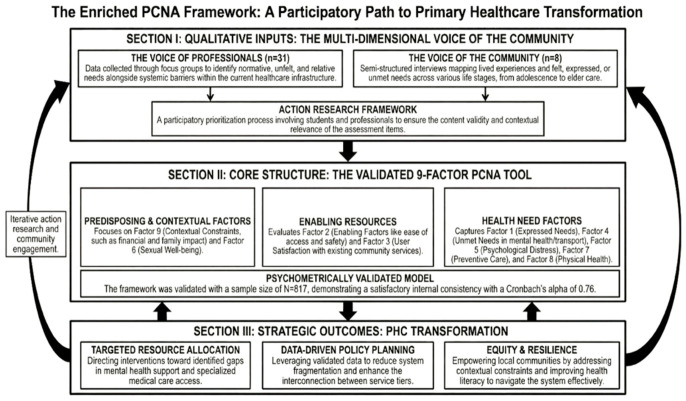
Overview of the PCNA development and validation framework. Section I illustrates the qualitative inputs; Section II presents the validated 9-factor structure organized by Andersen’s Behavioral Model dimensions; Section III outlines potential strategic applications for PHC planning, as discussed in the study implications. Note: Figure 1 was created by the authors with the assistance of AI-based tools for visualization purposes only.

The PCNA questionnaire was developed using a sequential mixed-methods design, integrating qualitative exploration into item generation alongside subsequent quantitative validation. The development process comprised three stages: (a) qualitative exploration of community healthcare needs through focus groups and interviews, (b) generation of questionnaire items informed by thematic analysis and alignment with Andersen’s Behavioral Model, and (c) psychometric evaluation of the resulting instrument. Mixed-methods approaches are widely recommended in instrument development, as they enable the integration of stakeholder-derived insights with statistical validation of measurement constructs [32,33]. The research architecture and the sequential steps of the study are visually summarized in Figure 1. Stages (a) and (b) drew on the same qualitative dataset but represent sequential analytical steps: stage (a) involved the open exploration and identification of community healthcare needs, while stage (b) involved the systematic translation of identified themes into candidate questionnaire items, guided by the dimensions of Andersen’s Behavioral Model [17,18].

#### 2.1.1. Qualitative Data Collection

The qualitative phase was conducted in Thessaloniki, Northern Greece, between May 2021 and June 2022, and comprised two complementary components: focus group discussions with PHC professionals and semi-structured interviews with community members.

Focus groups with PHC professionals: Three focus group discussions were conducted with a total of 31 PHC professionals, selected through purposive, theory-informed sampling. Eligibility criteria included current employment in PHC services or active involvement in clinical training related to PHC; individuals with no formal association to PHC were excluded.

Participants were recruited through professional health networks, postgraduate university programs in PHC, and direct contact with local PHC units.

The three groups were composed as follows: FG1 included fourteen postgraduate students from a multidisciplinary PHC program, whose inclusion captured early-career perspectives; FG2 involved eleven general medicine residents undergoing clinical training in PHC; and FG3 comprised six experienced professionals from an interdisciplinary team at a local PHC unit (TOMY) in Thessaloniki, including general practitioners, community nurses, a health visitor, and a social worker.

The first focus group was conducted online via Zoom due to COVID-19 restrictions, while the second and third were held in person—at a university facility and a local PHC unit, respectively. All sessions lasted 90–120 min, were facilitated by two experienced moderators (one from psychology and one from adult education), and were audio-recorded and video-recorded with participants’ written informed consent.

Discussions followed a semi-structured guide developed specifically for the study, covering perceived and unmet healthcare needs, healthcare-seeking behavior, service gaps, participatory planning, and PHC’s impact on community well-being. The guide was informed by international literature on needs assessment and aligned with the core dimensions of Andersen’s Behavioral Model (predisposing, enabling, and need-related factors). In line with the study’s exploratory design, insights from the first two groups informed iterative refinements to the discussion guide used in FG3.

Semi-structured interviews with community members: To incorporate the perspectives of potential service users, eight semi-structured interviews were conducted with adult community members representing different life stages (ages 15–67), including adolescents, young adults, a pregnant woman, middle-aged adults, and older adults.

These participants were not limited to current health service users but were selected purposively to capture variation in age, gender, and life-stage-specific healthcare needs.

The interviews were conducted by the first author at the participants’ homes, were audio-recorded, and were transcribed verbatim.

Interview guides were developed based on themes emerging from the preceding focus group analysis, enabling the exploration of expressed and perceived unmet needs, factors influencing access to care, and healthcare experiences from the community perspective.

The distinction between professional and community perspectives reflects a deliberate methodological choice: while PHC professionals offered insights into population needs as observed through service delivery (normative and comparative needs), community members articulated their own experiences, priorities, and self-identified needs (felt and expressed needs). Although both groups are part of the same PHC ecosystem, they provide complementary viewpoints that together inform a more comprehensive understanding of healthcare needs.

#### 2.1.2. Qualitative Data Analysis

All focus group sessions and individual interviews were transcribed verbatim. Transcription of focus group recordings was performed by the first author and reviewed for accuracy by a second researcher.

Focus group data were analyzed using Interpretative Phenomenological Analysis (IPA), following the eight-step framework proposed by Palmer et al. (2010) for focus group data [39]. This approach focused on identifying “objects of concern”—explicit needs, frustrations, and values expressed by participants—and analyzing participants’ roles, interactions, and use of language. Deductive coding aligned with the three core dimensions of Andersen’s Behavioral Model (predisposing, enabling, and need factors) was used alongside inductive codes emerging from the data. Emergent themes were revisited in light of group dynamics and refined through repeated engagement with the full transcripts.

Interview data were analyzed using thematic analysis, following the six-phase framework of Braun and Clarke (2006) [34]. Initial coding was performed by the first author, with subsequent review and discussion with a second researcher to reach consensus on the thematic structure.

The combined analysis yielded core thematic domains, including perceptions of health needs beyond clinical diagnoses, barriers to accessing care, the role of community context, trust and communication with providers, unexpressed and stigmatized needs, service fragmentation, and preventive care gaps. These domains—encompassing physical, psychological, social, and access-related dimensions—were subsequently mapped onto the predisposing, enabling, and need-related dimensions of Andersen’s Behavioral Model and translated into an initial pool of 65 candidate questionnaire items.

#### 2.1.3. Item Development and Origins

The majority of questionnaire items were originally developed through the qualitative phase of this study, directly derived from the themes identified in the focus groups and interviews. A subset of items was informed by domains covered in established instruments, including the Primary Care Assessment Tool (PCAT) [23], the Nottingham Health Profile (NHP) [22], ensuring alignment with recognized dimensions of healthcare needs assessment while maintaining the participatory orientation of the PCNA.

The preliminary version of the PCNA was reviewed by an expert panel comprising three PHC researchers and two practicing clinicians, who assessed items for clarity, relevance, and comprehensiveness. Cognitive interviews (*n* = 20) using a think-aloud protocol were subsequently conducted to identify ambiguous or problematic items. Refinements included rewording unclear items, adjusting response option labels for consistency, and reordering items to improve logical flow.

Following these steps, community engagement activities were conducted to further ensure contextual relevance and community acceptability. Six group sessions with community stakeholders—including a subset of original qualitative participants and additional community members—were held at community venues and focused on ranking and prioritizing item domains. Complementary individual sessions focused specifically on item clarity and wording. Participants included general practitioners, community nurses, and community members; terminology was standardized to “general practitioners” throughout, consistent with Greek PHC nomenclature.

The instrument was subsequently advanced to quantitative evaluation to examine its factorial structure and psychometric properties.

### 2.2. Participants and Procedure

A total of 1030 questionnaires were distributed through a combination of in-person and online data collection across community and PHC settings in the Thessaloniki metropolitan area and surrounding rural areas of Northern Greece. Of these, 817 were fully completed and included in the analysis (response rate: 79.3%). In-person data collection involved trained research assistants administering paper questionnaires face-to-face at recruitment sites; online completion was offered as an alternative for participants who preferred it. No incentives were provided for participation.

Community settings included public venues such as community centers, municipal offices, and public events, where non-service-users were also reached. PHC settings referred to primary care units where participants were recruited during or after visits. This distinction ensured that the sample captured both active service users and community members whose healthcare needs may remain unexpressed within formal healthcare encounters.

This community-embedded, multi-site recruitment strategy is consistent with international recommendations for participatory health needs assessment. The WHO has emphasized that effective PHC systems require the active engagement of communities in identifying health priorities and co-designing responses [30,35]. A strictly clinical or service-based recruitment approach would have limited the sample to active service users, thereby excluding individuals whose healthcare needs remain unmet or unexpressed—precisely the populations that participatory needs assessment aims to reach [13,36].

Recruitment was conducted in collaboration with municipal health departments, local community centers, patient associations, and rural PHC units. Diversity in the sample was promoted through targeted recruitment across age groups, gender, education levels, urban and rural residence, and chronic disease status. Descriptive statistics for the sample are reported in Table 1.

The pilot testing, cognitive interviews (*n* = 20), and community engagement activities that informed the refinement of the questionnaire prior to main data collection are described in Section 2.1.3.

### 2.3. PCNA Questionnaire

The PCNA is a multidimensional, self-administered questionnaire designed to assess perceived healthcare needs in PHC populations. The questionnaire captures a range of domains, including individual perceptions of health, access to services, psychosocial factors, and interaction with the health system, informed by Andersen’s Behavioral Model of Health Services Use. Accordingly, the questionnaire incorporates dimensions related to predisposing characteristics, enabling resources, and perceived needs, allowing for a comprehensive assessment of factors influencing health service utilization.

The PCNA questionnaire uses 5-point Likert-type response scales with domain-appropriate anchors (e.g., 1 = Very poor to 5 = Very good for health evaluations; 1 = Not at all to 5 = Very much for service utilization items). The initial questionnaire comprised 65 items covering sociodemographic characteristics, health status perceptions, service utilization, satisfaction, unmet needs, and psychosocial and contextual factors. Following pilot testing, participatory refinement, and iterative factor analysis (see Section 2.4), the instrument was reduced to 33 items through EFA (10-factor solution) and subsequently refined to 29 items through CFA (9-factor solution). The item reduction pathway is documented in Supplementary Table S3.

Participants in the quantitative phase completed the PCNA questionnaire (29 items plus a sociodemographic section) as a self-administered paper questionnaire or equivalent online form. No additional tasks beyond questionnaire completion were required.

The factorial structure and construct validity of the questionnaire were examined through exploratory factor analysis (EFA) and confirmatory factor analysis (CFA), while internal consistency was assessed using Cronbach’s alpha coefficients.

### 2.4. Statistical Analysis

Statistical analysis was conducted to examine the factorial structure, construct validity, and internal consistency of the PCNA questionnaire. Statistical significance was set at *p* < 0.05 for all analyses.

For psychometric validation, the total sample was randomly split into two independent subsamples for cross-validation purposes. The first subsample (*n* = 520) was used for exploratory factor analysis (EFA) to identify the underlying factor structure, and the second subsample (*n* = 297) for confirmatory factor analysis (CFA) to test the proposed measurement model. This random allocation approach ensured that the two subsamples were drawn from the same population under comparable data collection conditions, and their sociodemographic comparability was examined (Table 1).

EFA was performed on the first subsample using principal axis factoring, which does not assume multivariate normality. Oblique rotation (Promax) was applied, given the expected correlations among latent factors. The adequacy of the data for factor analysis was assessed using the Kaiser–Meyer–Olkin (KMO) measure of sampling adequacy and Bartlett’s test of sphericity. Factor retention was based on eigenvalues greater than 1.0, a widely used criterion in factor analytic research [40,41,42], inspection of the scree plot (Figure S1), and interpretability of the factor solution. An iterative item-reduction procedure was employed across eight successive steps: at each step, items with factor loadings below 0.40 or with cross-loadings ≥ 0.40 on more than one factor were removed, consistent with commonly applied thresholds in scale development [33], and the analysis was repeated on the remaining items until a stable and interpretable solution was obtained.

CFA was subsequently conducted on the second independent subsample to evaluate the fit of the proposed measurement model. Model fit was assessed using multiple indices, including the chi-square-to-degrees-of-freedom ratio (χ^2^/df), the Comparative Fit Index (CFI), the Tucker–Lewis Index (TLI), the Root Mean Square Error of Approximation (RMSEA), and the Standardized Root Mean Square Residual (SRMR). Acceptable model fit was determined based on commonly recommended thresholds (CFI and TLI ≥ 0.90, RMSEA ≤ 0.08, SRMR ≤ 0.08).

Internal consistency of the questionnaire and its subscales was assessed using Cronbach’s alpha coefficients.

All analyses were performed using IBM SPSS Statistics version 21.0 and AMOS version 18.0.

### 2.5. Ethics Approval and Consent to Participate

The study was conducted in accordance with established ethical standards for social research and complied with the principles of the American Psychological Association (APA) Code of Ethics. The study was approved by the Bioethics and Ethics Committee of the School of Medicine, Aristotle University of Thessaloniki (Approval No. 5.170/18.12.2019).

Written informed consent was obtained from all qualitative study participants (focus groups and individual interviews) prior to participation. For minor participants, written parental/guardian consent was obtained in all cases. For the quantitative component, participants completing paper questionnaires signed a Participant Information Sheet and Consent Form prior to questionnaire completion. For online participants, informed consent was obtained electronically: participants reviewed the study information sheet and indicated their consent by proceeding with questionnaire completion. No personally identifiable information was collected in either mode of administration.

Audio and video recordings from focus groups and audio recordings from individual interviews were collected solely for transcription and analysis purposes and were securely stored in accordance with data protection regulations.

## 3. Results

### 3.1. Qualitative Findings Informing Item Development

The qualitative phase comprised three focus groups with 31 PHC professionals and eight semi-structured interviews with community members representing different life stages (two adolescents aged 15 and 16; one pregnant woman aged 34; two working-age adults aged 37 and 48; and three older adults aged 52, 65, and 67). Data were analyzed using IPA for focus groups and thematic analysis for interviews, and mapped onto Andersen’s Behavioral Model dimensions. Five cross-cutting themes emerged across both data sources and informed the content of the PCNA questionnaire. A detailed mapping of qualitative themes to specific PCNA items is provided in Supplementary Table S2.

#### 3.1.1. Unexpressed and Unrecognized Healthcare Needs

The most pervasive finding across all eight interviews was the presence of healthcare needs that remained unexpressed, unrecognized, or unaddressed. This pattern manifested differently across life stages but was universally present. Adolescents described reluctance to disclose emotional difficulties: “I used to think it was unnecessary to burden my friends with such problems” (Int.1, F, 16). Similarly, a 15-year-old male reported: “I don’t discuss it easily with my parents because I feel they won’t quite understand or will make it a bigger deal” (Int.3, M, 15). Among working-age men, unexpressed needs were attributed to gendered norms: “I won’t say ‘I’m anxious’ or ‘I’m not well.’ You keep it inside. It’s perhaps a matter of mentality” (Int.7, M, 48). At mid-life, a 52-year-old woman described sexual health as a systematically silenced domain: “We don’t discuss these easily. Not always with the doctor, nor necessarily with the partner… So it stays inside you. And that is a need that is not met” (Int.5, F, 52). These findings directly informed the inclusion of items capturing self-perceived psychological well-being (B3), sexual well-being (B5, B7), and satisfaction with available services (B13), designed to elicit needs that would otherwise remain invisible to conventional clinical assessment.

#### 3.1.2. Subthreshold Psychological Distress Across the Life Course

All eight participants described psychological distress that fell below clinical thresholds but significantly affected daily functioning and quality of life. This was not episodic crisis but persistent, low-grade pressure. A 37-year-old father described it as: “It is not anxiety that overwhelms you, but it is constant. You think about work, money, family… there is a responsibility that never stops” (Int.8, M, 37). An older man articulated how fear, rather than symptoms, constituted the primary burden: “Is this normal? Or is it the beginning of something worse? It’s the fear that causes the anxiety, not the symptom itself” (Int.6, M, 67). During pregnancy, distress was experienced as episodic but intense: “It comes in waves. For example, before tests or when I think about the birth” (Int.4, F, 34). A 52-year-old woman described the emotional impact of life-stage transitions: “When the children leave, when the house empties… you feel it” (Int.5, F, 52). These accounts informed PCNA items on psychological well-being (B3), stress management (B4), and time management as a distress proxy (B6).

#### 3.1.3. Avoidance and Postponement of Preventive Care

Six of eight participants described patterns of avoiding or postponing preventive healthcare, driven by distinct but complementary mechanisms. A 48-year-old man acknowledged fear-based avoidance: “I avoid it a bit. If there is no reason, I don’t go. Perhaps because I don’t want to hear something” (Int.7, M, 48). A younger man framed it as competing priorities: “There is always something more urgent—work, family. And you say ‘I’m fine, I’ll do it later’” (Int.8, M, 37). A 52-year-old woman, by contrast, engaged in preventive screening but described its psychological cost: “Every time there is this anxiety until the results come out. You say, ‘what if something is found?’” (Int.5, F, 52). Among adolescents, a different pattern emerged—not avoidance but superficial engagement: “At school they say some things, but very generally. I don’t feel that if I had questions I could resolve them there” (Int.3, M, 15). A pregnant woman identified information overload as a barrier: “You read so much that you end up confused and more anxious. I would prefer more organized and reliable guidance” (Int.4, F, 34). These findings informed PCNA items on the perceived usefulness of preventive screening (B9), knowledge about preventive services (B10), and awareness of STIs and contraception (B8).

#### 3.1.4. Demand for Holistic PHC Beyond the Biomedical Model

Seven of eight participants articulated a need for healthcare that extends beyond clinical diagnosis and treatment. An older man expressed this most directly: “I would like a system that supports us more comprehensively—not only when we get sick” (Int.6, M, 67). A pregnant woman echoed this: “Pregnancy is a period when you need support on many levels, not just medical” (Int.4, F, 34). At mid-life, the gap between available and desired care was apparent: “Someone to talk to about the changes in your body, your psychology, your life. Because this age has many changes, but they are not addressed comprehensively” (Int.5, F, 52). Among adolescents, the demand was for meaningful rather than tokenistic support: “Yes, but it should be something meaningful. Not just someone coming once and saying general things” (Int.3, M, 15); “Every time a psychologist comes to talk to us, all they talk about is stress management—things a friend of mine could have told me” (Int.1, F, 16). A 37-year-old man articulated the need for proactive engagement: “Perhaps it would help if there were ways to mobilize you earlier, before it becomes a problem” (Int.8, M, 37). These perspectives informed the PCNA’s emphasis on perceived adequacy of PHC services (B13, B22–B24) and unmet needs across multiple domains (B26–B29).

#### 3.1.5. Financial and Family Context as Health Determinants

Contextual factors—particularly economic constraints and family roles—emerged as significant shapers of healthcare needs and access. A 65-year-old caregiver described the invisible burden of informal care: “I may have some professionals helping, but the worry and the thinking always fall on me. It always passes through me first” (Int.2, F, 65). Financial barriers were identified across ages: “We all want public primary healthcare to be available to everyone and as it should be” (Int.2, F, 65); “If something more serious is needed, what will I do? That is anxiety” (Int.6, M, 67). A pregnant woman experienced both constraints simultaneously: “We would like the public system to be able to offer the same level of care, without this financial burden” (Int.4, F, 34). Self-care was consistently deprioritized in favor of family obligations: “There ends up being no care for yourself” (Int.7, M, 48). These findings directly informed items on the impact of family (B11) and financial situation (B12) on health, as well as enabling factors related to service accessibility and affordability (B19–B21).

### 3.2. Sample Characteristics

The following results pertain to Step C (psychometric evaluation) of the study. The qualitative findings from Steps A and B are presented in Section 3.1.

The study sample consisted of 817 participants, with a mean age of 45.8 years (SD = 13.6). For the purposes of factor analysis, the sample was randomly divided into two subsamples: an exploratory factor analysis (EFA) subsample (*n* = 520; mean age = 46.1 years, SD = 14.1) and a confirmatory factor analysis (CFA) subsample (*n* = 297; mean age = 45.5 years, SD = 12.6). The two subsamples did not differ substantially in their sociodemographic characteristics.

The sample was predominantly female (71.8%). Most participants (61.3%) reported not having a chronic health condition, while a substantial proportion (38.7%) reported having one. A substantial proportion of participants (60.1%) had completed higher education.

Key sociodemographic characteristics of the total sample are presented in Table 1.

### 3.3. Exploratory Factor Analysis (EFA)

Exploratory factor analysis (EFA) was conducted on the first subsample (*n* = 520) to identify the underlying structure of the questionnaire. The initial analysis included 65 items. Sampling adequacy for the final solution was confirmed (KMO = 0.78), and Bartlett’s test of sphericity was statistically significant (χ^2^(528) = 6003.69, *p* < 0.001), supporting the suitability of the data for factor analysis (Table 2).

The exploratory factor analysis (EFA) initially revealed a multidimensional structure comprising 10 factors, accounting for 55.05% of the total variance, indicating a satisfactory representation of the underlying construct [32,33]. An iterative item reduction process was conducted based on factor loadings (<0.40) and substantial cross-loadings, resulting in a stable and interpretable factor solution. Sampling adequacy and suitability for factor analysis were confirmed (KMO = 0.78; Bartlett’s Test of Sphericity, *p* < 0.001).

The analysis was performed using principal axis factoring with Promax rotation. Factor retention was guided by eigenvalues greater than 1, scree plot inspection (Figure S1), and conceptual interpretability. The final solution retained 33 items loading onto 10 factors, corresponding to key conceptual domains, including expressed needs, reasons for accessing care, user satisfaction, unmet needs, psychological distress, sexual well-being, preventive care, physical health, contextual constraints, and rest-related concerns. This structure reflects the multidimensional nature of healthcare needs, encompassing individual, service-related, and contextual dimensions. The factorial structure identified through EFA was subsequently evaluated using confirmatory factor analysis (CFA) on an independent subsample.

### 3.4. Confirmatory Factor Analysis (CFA)

The EFA-derived structure was subsequently evaluated through CFA on an independent subsample (*n* = 297). Three competing measurement models were tested and compared (Table 3). Model 1 replicated the 10-factor, 33-item structure derived from EFA. Model 2 retained 10 factors but removed two items: Item 8 (economic reasons for PHC access), due to content overlap with Item 6 (free and public services), and Item 20 (mood fluctuations), due to ambiguous wording relative to the remaining items. Model 3 further excluded the rest/sleep factor (Items 32–33), as its two items showed substantial content overlap and the construct extended beyond the core domains targeted by the instrument, resulting in a 9-factor, 29-item structure (Table 4).

As shown in Table 3, Model 3 demonstrated the best fit to the data (χ2/df = 1.675, RMSEA = 0.048, CFI = 0.92, TLI = 0.90, SRMR = 0.06) and the lowest AIC (762.350), sup-porting its selection as the most parsimonious and theoretically coherent solution.

Standardized factor loadings for all retained items were statistically significant (*p* < 0.001), with the majority of items demonstrating moderate to strong loadings. One item (frequency of physical activity, λ = 0.35) showed a comparatively lower loading but was retained due to its conceptual relevance to the Physical Health domain and its contribution to the construct’s content coverage, consistent with established practices in scale development.

Detailed factor loadings are provided in Table S1 (Supplementary Materials). The CFA path diagram, including inter-factor correlations, is presented in Figure S2 (Supplementary Materials).

### 3.5. Reliability

The internal consistency of the PCNA questionnaire was assessed using Cronbach’s alpha coefficients. The overall scale demonstrated adequate to high internal consistency (α = 0.76), while subscale values ranged from 0.62 to 0.91.

Except for the physical health subscale (α = 0.62), all factors exceeded the commonly accepted threshold of 0.70. The lower value observed for the physical health factor remains acceptable in the context of early-stage instrument validation.

### 3.6. Descriptive Findings

Frequency distributions and percentages indicated that, despite generally positive self-rated physical health, a substantial proportion of participants reported difficulties related to sleep quality, stress, and psychological well-being. Preventive care was considered important, although gaps in awareness of available services were observed.

Moderate levels of satisfaction with health services were reported. Notably, significant unmet needs were identified across several service areas. Mental health services were the most frequently reported deficiency, followed by transportation services, access to specialist physicians (e.g., cardiologists, endocrinologists), and health information/preventive screenings (Table 5).

The final version of the PCNA questionnaire, including instructions, consent information, and all items, is provided in Appendix S1 (Supplementary Materials). A detailed mapping of qualitative interview themes to final PCNA items is provided in Supplementary Table S2, and the item reduction pathway is documented in Supplementary Table S3.

## 4. Discussion

The present study developed and validated the PCNA questionnaire using a mixed-methods approach integrating qualitative exploration into a sequential design comprising exploratory and confirmatory factor analysis. The findings support the factorial validity and internal consistency of the questionnaire, while demonstrating its capacity to capture the multidimensional and context-dependent nature of healthcare needs in PHC settings. These findings should be interpreted considering the limitations of existing health needs assessment approaches, which have been characterized by a predominant focus on biomedical indicators and limited integration of contextual and community-derived perspectives into assessment frameworks [2,6,8,43].

The factorial structure identified through EFA and confirmed through CFA reflects a multidimensional conceptualization of healthcare needs, encompassing not only individual health status and service utilization, but also psychological, social, and contextual dimensions. This is consistent with established models of health service utilization, particularly the Andersen Behavioral Model, which emphasizes the interaction between predisposing, enabling, and need-related factors [11,12,13,14,15].

Alignment of the PCNA Factor Structure with Andersen’s Behavioral Model: To further clarify the theoretical underpinnings of the PCNA factor structure, the nine validated factors can be explicitly mapped onto the three core dimensions of Andersen’s Behavioral Model of Health Services Use (Table 6). This mapping demonstrates that the instrument’s empirical structure aligns with its theoretical foundations, while also extending the model’s operationalization to include dimensions that are often underrepresented in existing PHC assessment tools.

As shown in Table 6, all three Andersen dimensions are represented in the final CFA model, with the nine PCNA factors distributing across them, though with a grader concentration in the need domain (five factors). This distribution reflects the instrument’s primary orientation toward capturing perceived and unmet healthcare needs, consistent with its design as a community-oriented needs assessment tool rather than a service performance measure. The predisposing dimension is represented by a single factor (Preventive Care Needs), capturing health beliefs and awareness that shape individuals’ propensity to engage with preventive services. Three factors map onto the enabling dimension: Enabling Factors (structural access), User Satisfaction (perceived system adequacy), and Contextual Constraints (family and financial determinants). These collectively operationalize the resources and circumstances that facilitate or hinder healthcare utilization.

Within the need dimension, the PCNA differentiates between physical health status (F8), psychological distress (F5), sexual well-being (F6), expressed needs translated into service utilization (F1), and unmet needs representing gaps between felt needs and available services (F4). This granular decomposition of the need construct is a distinctive feature of the PCNA: whereas many existing instruments treat “need” as a unitary construct, the PCNA reflects the qualitative finding that healthcare needs are multidimensional, life-stage-dependent, and often unexpressed (see Section 3.1).

This factor–theory alignment also provides a framework for interpreting the reduction from 10 (EFA) to 9 (CFA) factors. Factor 10 (Rest-related Concerns), which demonstrated marginal internal consistency (α = 0.62) and conceptual overlap with adjacent factors, was removed during CFA model refinement. This reduction is consistent with standard EFA-to-CFA refinement processes in scale development [44], where factors with borderline reliability and ambiguous theoretical positioning are consolidated. The removal of Factor 10 did not diminish the instrument’s theoretical coverage of Andersen’s three dimensions; rather, it improved model parsimony while retaining comprehensive domain representation. This refinement reflects both statistical optimization and clearer delineation of construct boundaries within primary health care needs.

Importantly, the integration of qualitative findings ensures that healthcare needs are operationalized not only as statistically derived constructs but also as lived and contextually embedded experiences, thereby strengthening the ecological validity of the instrument. In this study, needs emerged not as static or purely clinical conditions, but as dynamic constructs shaped by everyday life, social context, health literacy, and access to services, consistent with prior research highlighting the limitations of traditional needs assessment approaches based on predefined and predominantly biomedical categories of need [4,5,8].

Building on this, the PCNA addresses key limitations of existing needs assessment tools by incorporating both individual and contextual dimensions of healthcare needs, as well as perspectives derived from both community members and PHC professionals. Compared to established instruments such as the Primary Care Assessment Tool (PCAT) [30], the Patient-Centred Primary Care Measure (PCPCM) [32], the Quality and Outcomes Framework (QOF) [45], and the Primary Health Care Performance Initiative (PHCPI) [46], the PCNA places greater emphasis on perceived and unmet needs, as well as on subjective and contextual dimensions of health, rather than focusing primarily on service performance, clinical indicators, or patient satisfaction. While the PCPCM has advanced the assessment of relational and person-centered aspects of care, it remains primarily focused on care experiences rather than the broader spectrum of community-perceived and context-dependent healthcare needs [31,33].

Several quantitative instruments are condition-specific or embedded within large-scale survey frameworks, which may limit their flexibility for community-oriented assessments [18]. At the same time, the CFA results indicate model fit indices comparable to those reported for the PCAT and PCPCM [30,32], supporting the psychometric robustness of the PCNA. However, consistent with Andersen’s behavioral framework and recent methodological perspectives, measurement constructs and their associations may vary across populations and contexts, reinforcing the need for ongoing validation [15,31].

The factorial structure of the PCNA, encompassing domains such as expressed needs, unmet needs, psychological distress, preventive care, and contextual constraints, aligns with previous multidimensional approaches [47]. Notably, the prominence of unmet needs related to mental health, access to specialized care, and structural barriers observed in this study is consistent with recent quantitative evidence highlighting the role of enabling and contextual factors in shaping access to care in PHC systems [10].

A key contribution of this study lies in the participatory and action-oriented approach adopted during instrument development. The integration of community engagement activities, stakeholder involvement, and iterative feedback processes into the development procedure enhanced the ecological and content validity of the questionnaire, ensuring that the resulting items reflect real-world experiences and priorities. This participatory mixed-methods approach responds to calls in the literature for more inclusive and context-sensitive models of health needs assessment [22,26,43], and aligns with participatory health approaches that position communities as active contributors to knowledge production rather than passive recipients of care [25]. In this context, stakeholder engagement functioned not only as a mechanism for contextualization but also as an iterative refinement process incorporating elements of pre-testing procedures described in the scale development literature, whereby items are progressively clarified, revised, and aligned with participants’ lived experience [19,48]. This approach is further supported by recent community-based health needs assessment research, which emphasizes the iterative development of tools through stakeholder feedback and their role in identifying priority health issues grounded in local context [16].

The findings also have implications for PHC practice and policy. The results underscore the need to strengthen system responsiveness and coordination, particularly in addressing gaps in mental health care, access to specialized services, and structural constraints such as transportation. This interpretation is supported by cross-national evidence demonstrating that unmet healthcare needs are closely associated with structural and enabling factors, including income level, healthcare resource availability, and geographical accessibility [3], as well as by research highlighting the role of access barriers in shaping health service utilization within PHC systems [12]. The findings further resonate with evidence from the Greek context, which underscores the need for more systematic, coordinated, and participatory approaches to health needs assessment [21].

Moreover, the findings support the view that needs assessment should not be treated as a one-time measurement process, but as an ongoing, collaborative practice embedded within PHC systems [23].

Several aspects of the psychometric findings warrant further discussion. The Physical Health subscale demonstrated a lower internal consistency (α = 0.62) compared to other factors, which may reflect the heterogeneity of the three items comprising this factor (dietary habits, general health, physical activity), each tapping a distinct behavioral domain. This interpretation is further supported by the comparatively lower CFA loading observed for the physical activity item, suggesting that while physical activity is conceptually integral to physical health status, it may function as a more distal indicator compared to dietary habits and self-rated health. While values above 0.60 are considered acceptable in early-stage instrument development [48], future revisions may benefit from expanding or refining this subscale. Beyond the Physical Health subscale, two additional factors warrant comment regarding their structure. While two-item factors may be considered a structural limitation, both the Sexual Well-being and Contextual Constraints factors demonstrated high internal loadings and strong theoretical grounding within Andersen’s framework. Their retention was guided by conceptual relevance rather than purely statistical criteria, consistent with recommendations for early-stage instrument development where theoretical coherence is prioritized alongside empirical fit [44]. Future studies with larger and more diverse samples should examine the stability and potential expansion of these factors. Overall, the PCNA contributes a multidimensional and participatory framework for assessing healthcare needs in primary care, bridging the gap between theoretical models and real-world experiences. By integrating subjective, contextual, and system-level dimensions, the instrument provides a foundation for more responsive, equitable, and community-oriented primary health care planning.

### 4.1. Limitations

A number of limitations should be considered when interpreting the findings of this study. First, the non-probabilistic, community-based recruitment strategy limits the generalizability of the results, although it was consistent with the participatory orientation of the study and aimed to enhance ecological validity. The diversity of recruitment settings and the combination of in-person and online data collection partially mitigate this limitation. Future studies should validate the instrument in more diverse populations and across different healthcare system contexts.

Second, the predominantly healthy sample may have limited the identification of healthcare needs associated with chronic conditions, suggesting the need for further validation in clinical populations. Third, the cross-sectional design precludes assessment of temporal stability. Fourth, the use of self-reported data may introduce response biases, including social desirability and recall bias [49]. Finally, although the sample was randomly split for EFA and CFA, temporal confounding cannot be entirely excluded.

The present study represents an initial phase of psychometric validation, primarily focused on factorial structure and internal consistency. Face and content validity were supported through expert review and cognitive interviews (*n* = 20), alongside qualitative-to-quantitative item development grounded in Andersen’s Behavioral Model. Construct validity was examined through EFA and CFA, demonstrating acceptable model fit (Table 6), and internal consistency was within acceptable ranges (overall α = 0.76). However, additional psychometric properties, including convergent and discriminant validity, criterion validity, and test–retest reliability, were not assessed and should be addressed in future research. Specifically, future studies should examine composite reliability and average variance extracted for convergent validity and assess temporal stability through test–retest designs.

This staged validation approach is consistent with recommended scale development practices, where establishing a stable factor structure precedes more advanced psychometric testing [19,44,48]. The life-stage-specific modules developed during the qualitative phase also warrant further targeted validation.

### 4.2. Strengths

The study presents several notable strengths. The development of the PCNA questionnaire was grounded in a sequential mixed-methods design integrating qualitative and quantitative approaches, allowing for both conceptual depth and empirical validation. The qualitative phase drew on two complementary data sources—focus groups with 31 PHC professionals and semi-structured interviews with eight community members across different life stages (ages 15–67)—ensuring that item development reflected both normative and felt healthcare needs. The systematic mapping of qualitative themes to final questionnaire items (Supplementary Table S2) and the documented item reduction pathway (Supplementary Table S3) provide a transparent audit trail from community voices to validated instrument.

The use of independent subsamples for exploratory and confirmatory factor analysis enhanced the robustness of psychometric evaluation. The explicit alignment of the 9-factor structure with Andersen’s Behavioral Model (Table 6) demonstrates theoretical coherence alongside empirical fit.

In addition, the participatory and community-informed approach adopted during instrument development strengthened the content and ecological validity of the questionnaire by aligning measurement with lived experience and stakeholder priorities. Notably, participants were not limited to health service users but included community members recruited from diverse social settings, enabling the assessment of healthcare needs beyond the boundaries of formal service utilization. This approach is aligned with the WHO Astana Declaration’s emphasis on empowering communities to identify and prioritize their own health needs [50].

The PCNA’s capacity to capture unexpressed and context-dependent needs—as evidenced by the qualitative findings (Section 3.1) and confirmed through its factorial structure—addresses a recognized gap in existing PHC assessment tools, which have predominantly focused on service performance and clinical indicators rather than community-perceived healthcare needs.

## 5. Conclusions

The present study aimed to develop and validate the PCNA questionnaire, a multidimensional and context-sensitive instrument for assessing perceived, unmet, and context-dependent healthcare needs in community populations within PHC settings. The findings demonstrate that the PCNA exhibits a stable factorial structure and satisfactory internal consistency, supporting its use as a reliable tool for assessing population healthcare needs.

By integrating quantitative validation with community-informed qualitative insights into a unified instrument development framework, the PCNA extends existing approaches to needs assessment beyond predominantly biomedical and service-centered models toward a more comprehensive understanding of healthcare needs. The instrument captures not only individual health status and service utilization, but also psychosocial and contextual dimensions, reflecting the complex interplay between individuals, health systems, and broader social determinants.

The participatory and action-oriented approach adopted in its development enhances the ecological validity and practical relevance of the questionnaire, supporting its use in evidence-informed planning, resource allocation, and service design within PHC systems.

In clinical practice, the PCNA can support PHC teams in systematically identifying unmet healthcare needs that extend beyond the clinical encounter—including unexpressed psychological distress, barriers to preventive care, and contextual constraints related to family roles and financial circumstances. Its application may inform targeted service planning, facilitate community participation in priority-setting, and contribute to more equitable resource allocation. The instrument may also serve as a monitoring tool for evaluating the impact of PHC reforms on population-perceived needs, and for identifying emerging healthcare needs across different life stages and community contexts.

Overall, the PCNA offers a structured and adaptable framework for assessing healthcare needs at the community level, shifting needs assessment from a measurement exercise to a participatory process embedded in PHC systems. Its application in practice may facilitate the alignment of services with community-identified needs and contribute to more responsive, equitable, and person-centred primary health care.

## Figures and Tables

**Table 1:** Sociodemographic characteristics of the sample (*n* = 817).

Variable	*n* (%)/Mean (SD)
Gender	
Male	230 (28.2)
Female	587 (71.8)
Age (years)	45.8 (13.6)
Education level	
Higher education	490 (60.1)
Lower education	327 (39.9)
Chronic health condition	
Yes	316 (38.7)
No	501 (61.3)
No response	8 (1.0)

Note: Values are presented as mean (SD) or *n* (%). Percentages are based on valid responses.

**Table 2:** Exploratory Factor Analysis (EFA) Results: Items, Means (SD), and Factor Loadings (*n* = 520).

Item	Description	Mean (SD)	F1 (α = 0.80)	F2 (α = 0.85)	F3 (α = 0.82)	F4 (α = 0.74)	F5 (α = 0.71)	F6 (α = 0.91)	F7 (α = 0.70)	F8 (α = 0.62)	F9 (α = 0.75)	F10 (α = 0.62 *)
1	For what reasons did you use primary health care services in the past year? [For diagnosis and specialized examinations]	2.37 (1.46)	0.88									
2	For what reasons did you use primary health care services in the past year? [For referral to specialist physicians]	2.02 (1.36)	0.75									
3	For what reasons did you use primary health care services in the past year? [For prescription of medications]	2.88 (1.57)	0.61									
4	For what reasons did you use primary health care services in the past year? [For management of chronic conditions (e.g., diabetes, cardiovascular diseases, respiratory diseases, etc.)]	1.76 (1.28)	0.57									
5	For what reasons did you use primary health care services in the past year? [For routine health check-ups and vaccinations]	3.16 (1.41)	0.42									
6	For what reasons did you use primary health care services in the past year? [For free public services]	2.71 (1.53)		0.88								
7	For what reasons did you use primary health care services in the past year? [Due to ease of access]	2.39 (1.42)		0.85								
8	For what reasons did you use primary health care services in the past year? [For financial reasons]	2.76 (1.56)		0.81								
9	For what reasons did you use primary health care services in the past year? [Because I feel familiarity and safety]	2.14 (1.36)		0.57								
10	Are you satisfied with the healthcare system in your community?	2.52 (1.13)			0.85							
11	Do you believe you have adequate access to healthcare when you need it?	2.81 (1.14)			0.80							
12	Is there an adequate number of health and social care services in your community?	2.62 (1.10)			0.74							
13	To what extent do the services provided by primary health care facilities meet your needs?	2.77 (1.12)			0.54							
14	In relation to your health and quality of life, which services do you believe are lacking in primary health care in your area? [Mental health services]	4.05 (1.10)				0.80						
15	In relation to your health and quality of life, which services do you believe are lacking in primary health care in your area? [Medical specialties]	3.76 (1.13)				0.67						
16	In relation to your health and quality of life, which services do you believe are lacking in primary health care in your area? [Transportation services to healthcare facilities]	3.55 (1.28)				0.64						
17	In relation to your health and quality of life, which services do you believe are lacking in primary health care in your area? [Health information/preventive screening services]	3.63 (1.21)				0.61						
18	How do you evaluate your ability to manage stress in your daily life?	3.27 (0.86)					0.78					
19	How would you describe your psychological well-being?	3.58 (0.82)					0.69					
20	Have you noticed fluctuations in your mood recently?	2.85 (1.25)					−0.47					
21	How do you evaluate your ability to manage your time between work, family, and personal time?	3.30 (0.93)					0.45					
22	How do you evaluate your sexual life?	3.18 (1.14)						0.95				
23	Are you satisfied with your sexual life?	3.11 (1.25)						0.90				
24	How informed do you feel about preventive health screenings (e.g., breast self-examination, Pap test, prostate screening, etc.)?	4.21 (1.01)							0.90			
25	Do you consider regular preventive screening to be useful?	4.54 (0.78)							0.58			
26	How informed do you feel about sexually transmitted infections and contraception?	4.14 (1.04)							0.50			
27	How do you evaluate your dietary habits in relation to your overall health?	3.58 (0.86)								0.73		
28	How do you evaluate your overall physical health?	3.84 (0.79)								0.72		
29	How often do you engage in physical activity?	3.26 (1.12)								0.46		
30	To what extent does your financial situation negatively affect your health?	2.90 (1.39)									0.77	
31	To what extent does your family situation negatively affect your health?	2.74 (1.38)									0.74	
32	Do you experience difficulties related to the quality of your sleep?	2.68 (1.56)										0.78
33	How do you evaluate the quality of your sleep and rest?	3.29 (0.88)										−0.59

Notes: Extraction Method: Principal Axis Factoring. Rotation Method: Promax. KMO = 0.78; Bartlett’s Test of Sphericity: χ^2^(528) = 6003.69, *p* < 0.001. Total variance explained = 55.05%. Only factor loadings ≥ 0.40 are presented; blank cells indicate loadings below the threshold. α = Cronbach’s alpha. * Factor 10 (Rest-related Concerns) was excluded from the final CFA model (see Section 3.3). Factors: F1 = Expressed Needs; F2 = Enabling Factors; F3 = User Satisfaction; F4 = Unmet Health Needs; F5 = Psychological Distress; F6 = Sexual Well-being; F7 = Preventive Care Needs; F8 = Physical Health Status; F9 = Contextual Constraints; F10 = Rest-related Concerns.

**Table 3:** Confirmatory Factor Analysis: Comparison of Three Competing Models (*n* = 297).

Model	χ^2^	df	χ^2^/df	CFI	TLI	RMSEA	SRMR	AIC
Model 1 (10 factors, 33 items)	881.81	450	1.960	0.88	0.86	0.057	0.067	1169.81
Model 2 (10 factors, 31 items)	654.28	389	1.682	0.91	0.90	0.048	0.061	930.28
**Model 3 (9 factors, 29 items)**	**574.35**	**341**	**1.675**	**0.92**	**0.90**	**0.048**	**0.060**	**762.35**

Notes: χ^2^ = chi-square goodness of fit; df = degrees of freedom; CFI = Comparative Fit Index; TLI = Tucker–Lewis Index; RMSEA = Root Mean Square Error of Approximation; SRMR = Standardized Root Mean Square Residual; AIC = Akaike Information Criterion. Model 3 (bold) is the selected model.

**Table 4:** Structure of the Validated 9-Factor, 29-Item PCNA Model (CFA, Model 3).

Latent Factors (Circles)	Observed Items (Rectangles)
F1: Expressed Needs	Items 1, 2, 3, 4, 5
F2: Enabling Factors	Items 6, 7, 9 (EFA Item 8 removed for redundancy with Item 6)
F3: User Satisfaction	Items 10, 11, 12, 13
F4: Unmet Health Needs	Items 14, 15, 16, 17
F5: Psychological Distress	Items 18, 19, 21 (EFA Item 20 removed for ambiguous wording)
F6: Sexual Well-being	Items 22, 23
F7: Preventive Care Needs	Items 24, 25, 26
F8: Physical Health Status	Items 27, 28, 29
F9: Contextual Constraints	Items 30, 31

Model Fit Indices: χ^2^/df: 1.675; RMSEA (Root Mean Square Error of Approximation): 0.048; CFI (Comparative Fit Index): 0.92; TLI (Tucker–Lewis Index): 0.90; SRMR (Standardized Root Mean Square Residual): 0.06.

**Table 5:** Community Perceptions of Deficiency in PHC Services.

Service Category (Lacking “Very Much”)	Percentage (%)
Mental Health Services	41.0%
Transportation Services to care facilities	28.2%
Medical Specialties	27.9%
Health Information/Preventive Screenings	26.4%

**Table 6:** Mapping of the 9-factor PCNA structure onto Andersen’s Behavioral Model dimensions.

Andersen Dimension	PCNA Factor	Items	Conceptual Alignment
Predisposing	F7: Preventive Care Needs	B8, B9, B10	Health beliefs, knowledge about prevention, and attitudes toward screening—characteristics that predispose individuals to seek or avoid care
Enabling	F2: Enabling Factors	B19, B20, B21	Structural and relational enablers: familiarity with services, availability of free/public care, ease of access
Enabling	F3: User Satisfaction	B13, B22, B23, B24	Perceived adequacy of the healthcare system as an enabling resource—satisfaction as facilitator or barrier to continued use
Enabling	F9: Contextual Constraints	B11, B12	Family and financial circumstances that shape the capacity to access and benefit from care
Need	F8: Physical Health Status	B1, B2, B25	Self-perceived physical health and functional capacity—the subjective component of perceived need
Need	F5: Psychological Distress	B3, B4, B6	Perceived psychological well-being, stress management, and time pressure—the psychological dimension of health needs
Need	F6: Sexual Well-being	B5, B7	Self-assessed sexual health and satisfaction—a dimension rarely included in PHC needs assessment tools
Need	F1: Expressed Needs	B14–B18	Reasons for actively seeking PHC services—need translated into healthcare-seeking behavior
Need	F4: Unmet Health Needs	B26–B29	Perceived gaps between felt needs and available services—the discrepancy between need and utilization

## Data Availability

The data presented in this study are available on reasonable request from the corresponding author. The data are not publicly available due to ethical restrictions.

## Supplementary Materials

The following supporting information can be downloaded at https://www.mdpi.com/article/10.3390/healthcare14101302/s1. Figure S1: Scree plot of eigenvalues from the final exploratory factor analysis (*n* = 520). The plot supports the retention of 10 factors, with eigenvalues leveling off after the tenth component. Table S1: Standardized factor loadings for the 9-factor, 29-item PCNA model (CFA, Model 3; *n* = 297). Figure S2: Confirmatory factor analysis path diagram for Model 3 (9-factor, 29-item PCNA), including standardized factor loadings and inter-factor correlations (n = 297). Model fit: χ2/df = 1.675, RMSEA = 0.048, CFI = 0.92, TLI = 0.90, SRMR = 0.060. Table S2: Mapping of Community Interview Data to the Final 29-Item PCNA Questionnaire (9-factor model). Table S3: Item Reduction Pathway: From Qualitative Themes to Validated Instrument. Appendix S1: The final version of the Primary Care Needs Assessment (PCNA) Questionnaire.

## Abbreviations

The following abbreviations are used in this manuscript:

AIC	Akaike Information Criterion
AVE	Average Variance Extracted
CBPR	Community-Based Participatory Research
CFI	Comparative Fit Index
CFA	Confirmatory Factor Analysis
CR	Composite Reliability
EFA	Exploratory Factor Analysis
HNA	Health Needs Assessment
HTMT	Heterotrait–Monotrait Ratio
ICC	Intraclass Correlation Coefficient
IPA	Interpretative Phenomenological Analysis
KMO	Kaiser–Meyer–Olkin
NHP	Nottingham Health Profile
PCAT	Primary Care Assessment Tool
PCNA	Primary Care Needs Assessment
PCPCM	Patient-Centred Primary Care Measure
PHC	Primary Health Care
RMSEA	Root Mean Square Error of Approximation
SRMR	Standardized Root Mean Square Residual
TLI	Tucker–Lewis Index
