# Supplementary material for: Development and Validation of the Primary Care Needs Assessment (PCNA) Questionnaire: A Participatory Multidimensional Approach to Identifying Health Needs
Source: Healthcare (Basel). 2026 May 11;14(10):1302. doi: 10.3390/healthcare14101302 (PMC13206681; doi:10.3390/healthcare14101302)
Supplement: Supplementary file 1 [file healthcare-14-01302-s001.zip › healthcare-4275687-supplementary.pdf]

**Supplementary Materials**

Development and Validation of the Primary Care Needs Assessment (PCNA) Questionnaire: A Participatory Multidimensional Approach to Identifying Health Needs

**Figure S1.** Scree plot of eigenvalues from the final exploratory factor analysis (n = 520). The plot supports the retention of 10 factors, with eigenvalues leveling off after the tenth component.

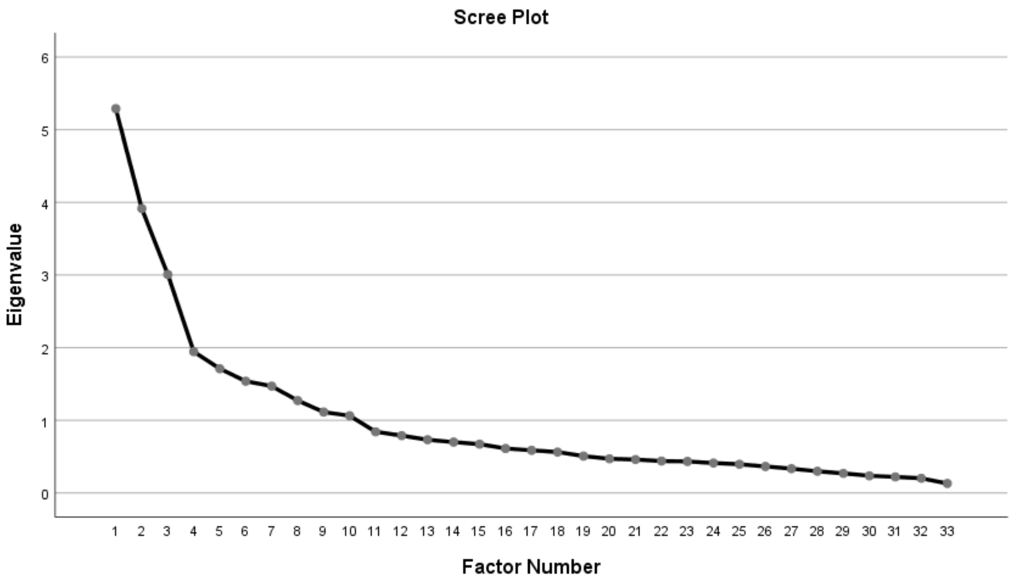


**Table S1:** Standardized factor loadings for the 9-factor, 29-item PCNA model (CFA, Model 3; n = 297).

| Factor | Item | Item Description | λ |
|---|---|---|---|
| **F1: Expressed Needs** | 1 | Diagnosis and specialized tests | 0.81 |
| | 2 | Referral to specialized medical specialties | 0.79 |
| | 3 | Medication prescription | 0.62 |
| | 4 | Management of chronic diseases | 0.58 |
| | 5 | Regular medical check-ups and vaccinations | 0.47 |
| **F2: Enabling Factors** | 6 | Free and public services | 0.71 |
| | 7 | Easy access | 0.87 |
| | 9 | Feeling of familiarity and safety | 0.76 |
| **F3: User Satisfaction** | 10 | Satisfaction with the healthcare system in the community | 0.92 |
| | 11 | Sufficient access to healthcare when needed | 0.69 |
| | 12 | Sufficient number of health and social services in the community | 0.67 |
| | 13 | Responsiveness of PHC services to personal needs | 0.63 |
| **F4: Unmet Health Needs** | 14 | Missing services: Mental health services | 0.70 |
| | 15 | Missing services: Medical specialties | 0.46 |

| Factor | Item | Item Description | λ |
|---|---|---|---|
| | 16 | Missing services: Transportation services to care facilities | 0.60 |
| | 17 | Missing services: Health information/preventive exams | 0.63 |
| **F5: Psychological Distress** | 18 | Evaluation of stress management in daily life | 0.64 |
| | 19 | Evaluation of psychological mood | 0.83 |
| | 21 | Evaluation of time management (work/family/self) | 0.61 |
| **F6: Sexual Well-being** | 22 | Evaluation of sexual life | 0.95 |
| | 23 | Satisfaction with sexual life | 0.88 |
| **F7: Preventive Care Needs** | 24 | Level of information regarding preventive examinations | 0.87 |
| | 25 | Perceived usefulness of regular preventive screenings | 0.54 |
| | 26 | Level of information on STDs and contraception | 0.56 |
| **F8: Physical Health Status** | 27 | Evaluation of dietary habits | 0.68 |
| | 28 | Evaluation of general physical health | 0.79 |
| | 29 | Frequency of physical activity | 0.35 |
| **F9: Contextual Constraints** | 30 | Negative impact of financial status on health | 0.78 |
| | 31 | Negative impact of family status on health | 0.82 |

**Notes:** λ = standardized factor loading. Extraction method: Maximum Likelihood estimation using AMOS 18.0. All loadings statistically significant at p <0.001. Item numbers correspond to the final 29-item PCNA instrument (see Appendix S1). Items 8 and 20 were excluded from the final model (see Section 3.3). Factor 10 (Rest-related Concerns, Items 32–33) was excluded from Model 3.

**Figure S2.** Confirmatory factor analysis path diagram for Model 3 (9-factor, 29-item PCNA), including standardized factor loadings and inter-factor correlations ($n$ = 297). Model fit: $\chi^2$/df = 1.675, RMSEA = 0.048, CFI = 0.92, TLI = 0.90, SRMR = 0.060.

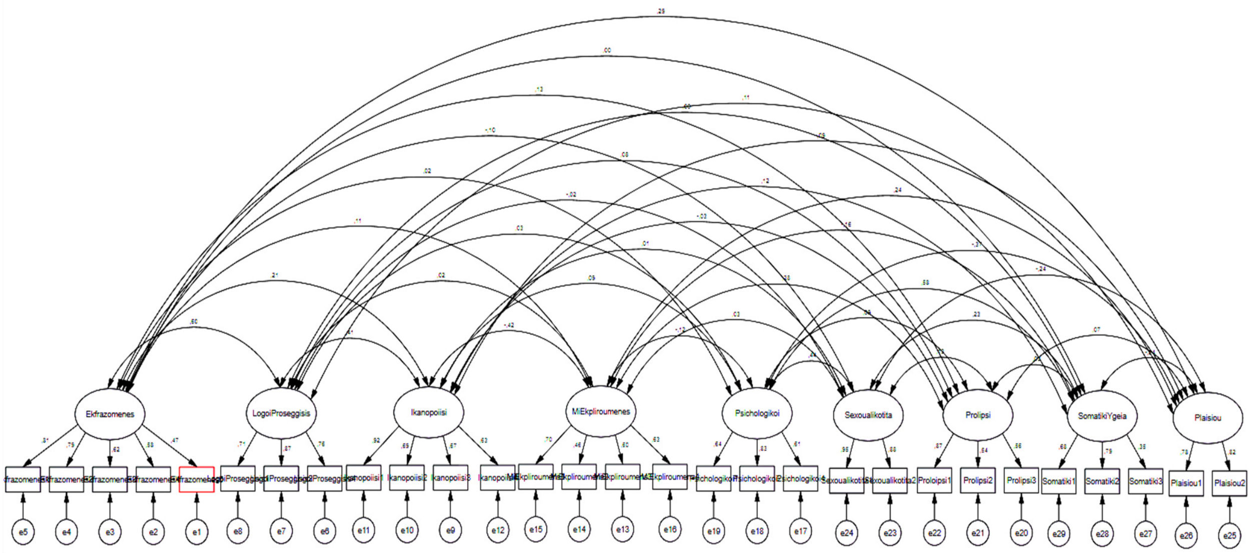

**Table S2:** Mapping of Community Interview Data to the Final 29-Item PCNA Questionnaire (9-factor model)

This table illustrates how themes from semi-structured interviews with eight community members informed the content of the final PCNA instrument. Interviews were conducted with participants representing different life stages: two adolescents (F, 16; M, 15), one pregnant woman (F, 34), two working-age adults (M, 37; M, 48), and three older adults (F, 52; F, 65; M, 67). Data were analyzed using thematic analysis (Braun & Clarke, 2006) and mapped onto the nine validated PCNA factors aligned with Andersen's Behavioral Model. The initial 65-item pool included life-stage-specific sections (pregnancy, adolescence, parenting, infant care) that were not retained; the general items (B1–B29) captured cross-demographic needs applicable across all life stages.

| Factor | Source(s) | Qualitative Theme | Item(s) | Rationale for Item |
|---|---|---|---|---|
| **F8: Physical Health Status (B1, B2, B25) - Andersen: Need** | | | | |
| **F8** | Int.2 (F,65); Int.6 (M,67) | *Age-related physical decline experienced as daily uncertainty: 'You wake up and say, how will I be today?' (Int.6); 'My stamina is not the same, and that suffocates me' (Int.2)* | **B1, B2** | Subjective health assessment shaped by felt decline, not diagnosis alone |
| **F8** | Int.5 (F,52) | *Body awareness changes at midlife: 'Before I never thought about it. Now I think about it all the time'; weight gain as 'losing control of my body'* | **B1, B2** | Transition from unreflective health to constant self-monitoring |
| **F8** | Int.1 (F,16); Int.3 (M,15) | *Adolescent health concerns tied to appearance: acne, weight fluctuations, body comparison with peers in sports team* | **B1, B2, B25** | Adolescent health perceived through body image, not clinical indicators |
| **F8** | Int.7 (M,48); Int.8 (M,37) | *Exercise intention-action gap: 'I don't do it. And I say it with disappointment' (Int.7); inconsistent exercise due to time/energy (Int.8)* | **B25** | Physical activity as unmet self-care need across working-age men |
| **F5: Psychological Distress (B3, B4, B6) - Andersen: Need** | | | | |
| **F5** | Int.7 (M,48) | *Chronic low-level anxiety: 'It is not anxiety that overwhelms you, but it is constant'; unexpressed distress: 'You keep it inside. It's perhaps a matter of mentality'* | **B3, B4** | Subthreshold distress invisible to clinical assessment |
| **F5** | Int.8 (M,37) | *Constant responsibility: 'Work, money, family… a responsibility that never stops'* | **B3, B4** | Young adult male distress driven by concurrent demands |
| **F5** | Int.1 (F,16); Int.3 (M,15) | *Academic anxiety: 'I reassure myself I still have time' (Int.1); avoidance coping: 'I don't sit down to process it much' (Int.3)* | **B3, B4** | Adolescent stress with avoidance as dominant strategy |
| **F5** | Int.6 (M,67) | *Fear of cognitive decline: 'Is this normal? Or the beginning of something worse? The fear causes the anxiety, not the symptom itself'* | **B3, B4** | Anticipatory anxiety in ageing, fear as unrecognized need |
| **F5** | Int.5 (F,52) | *Empty nest distress: 'When the children leave, when the house empties… you feel it'* | **B3** | Life-stage-specific psychological need, existential, not clinical |
| **F5** | Int.4 (F,34) | *Pregnancy anxiety 'in waves': before tests, thinking about birth, fear of maternal competence* | **B3, B4** | Episodic anticipatory distress distinct from chronic anxiety |
| **F5** | Int.7 (M,48); Int.5 (F,52); Int.8 (M,37) | *Self-care deprioritized: 'There ends up being no care for yourself' (Int.7); 'The self is always last' (Int.5); 'Yes, but not now' (Int.8)* | **B6** | Time management as distress proxy across 37-52 age range |
| **F5** | Int.1 (F,16); Int.3 (M,15) | *Difficulty expressing emotions: 'I thought it unnecessary to burden my friends' (Int.1); 'Parents won't understand or will make it bigger' (Int.3)* | **B3** | Unexpressed needs in adolescence across both genders |
| **F6: Sexual Well-being (B5, B7) - Andersen: Need** | | | | |
| **F6** | Int.5 (F,52) | *Sexual health as silenced need: 'We don't discuss these easily. Not with the doctor nor the partner… it stays inside you. That is a need that is not met'* | **B5, B7** | Strongest justification for F6, sexual needs systematically unaddressed |
| **F6** | Int.7 (M,48) | *Declining interest from fatigue/stress: 'It is not something you discuss. You set it aside. As if it were not a priority'* | **B5, B7** | Male perspective mirrors female: sexual health deprioritized |
| **F6** | Int.8 (M,37) | *Sexual life 'good but affected by daily rhyth, not a health issue, more a rhythm-of-life issue'* | **B5, B7** | Sexual well-being as lifestyle indicator, not clinical complaint |
| **F7: Preventive Care Needs (B8, B9, B10) - Andersen: Predisposing** | | | | |
| **F7** | Int.1 (F,16); Int.3 (M,15) | *STI knowledge gaps: 'very general' school info; 'I could not resolve my questions there' (Int.3); HPV vaccine for boys unknown (Int.1)* | **B8** | Health literacy gaps despite multiple information sources |
| **F7** | Int.7 (M,48) | *Avoidance of screening: 'I avoid it. Perhaps because I don't want to hear something'* | **B9, B10** | Fear as emotional barrier to preventive behaviour |
| **F7** | Int.5 (F,52) | *Screening with anxiety: 'Every time there is anxiety until the results come out. You say, what if something is found?'* | **B9, B10** | Even when screening occurs, psychological cost accompanies it |
| **F7** | Int.8 (M,37) | *Prevention postponed: 'There is always something more urgent. You say, I'm fine, I'll do it later'* | **B9, B10** | Competing demands crowd out prevention |

| | | | | |
|---|---|---|---|---|
| **F7** | Int.4 (F,34) | *Information overload: 'You read so much you end up confused. I would prefer more organized guidance'* | **B9, B10** | Health literacy ≠ information volume, need for structured guidance |
| **F9: Contextual Constraints (B11, B12) - Andersen: Enabling** | | | | |
| **F9** | Int.2 (F,65) | *Caregiver burden: 'The worry always falls on me. It always passes through me first'* | **B11** | Family role as invisible health determinant |
| **F9** | Int.6 (M,67) | *Financial insecurity: 'If something more serious is needed, what will I do? That is anxiety'* | **B12** | Economic constraint as anticipatory health anxiety |
| **F9** | Int.4 (F,34) | *Balancing child/work/self; financial cost of private birth: 'We would like the public system to offer the same level'* | **B11, B12** | Both contextual constraints active during pregnancy |
| **F9** | Int.1 (F,16); Int.3 (M,15) | *Divorced parents (Int.1); bullying climate: 'You try not to stand out or give cause' (Int.3)* | **B11** | Social context shapes adolescent well-being beyond clinical scope |
| **F1: Expressed Needs (B14 – B18) - Andersen: Need** | | | | |
| **F1** | Int.2 (F,65); Int.6 (M,67) | *Active PHC use for chronic disease, medication, checkups; medication dependency: 'it's like a door opening' (Int.6)* | **B14-B17** | Elderly utilization pattern, chronic management dominates |
| **F1** | Int.1 (F,16); Int.3 (M,15) | *Minimal PHC contact: vaccinations, prescriptions, sports certificates only* | **B14, B18** | Adolescent disengagement, reactive, not proactive |
| **F2: Enabling Factors (B19 – B21) - Andersen: Enabling** | | | | |
| **F2** | Int.2 (F,65); Int.6 (M,67) | *Desire for public PHC: 'We all want public primary care available to everyone' (Int.2); system difficulties (Int.6)* | **B20, B21** | Access and affordability as key enabling factors for older adults |
| **F2** | Int.4 (F,34); Int.1 (F,16) | *Private sector for safety/speed; 'We would like the public system without this financial burden' (Int.4)* | **B19-B21** | Trust, familiarity, and cost shape healthcare choices across ages |
| **F3: User Satisfaction (B13, B22 – B24) - Andersen: Enabling** | | | | |
| **F3** | Int.6 (M,67) | *Wants proactive system: 'A system that supports us comprehensively, not only when we get sick'; independence as central concern* | **B13, B22-B24** | Satisfaction tied to systemic adequacy, not encounters |
| **F3** | Int.1 (F,16); Int.3 (M,15) | *School support dismissed: 'What they tell us is something a friend could have told me' (Int.1); 'It should be something meaningful' (Int.3)* | **B13, B23** | Youth dissatisfaction, demand for substantive support |
| **F3** | Int.5 (F,52) | *Closing: 'Someone to talk to about changes in body, psychology, life. This age has many changes but not addressed comprehensively'* | **B13, B22, B24** | Mid-life demand for holistic PHC beyond screening |
| **F3** | Int.4 (F,34) | *Closing: 'Pregnancy is a period when you need support on many levels, not just medical'* | **B13** | PHC adequacy measured against holistic expectations |
| **F4: Unmet Health Needs (B26 – B29) - Andersen: Need** | | | | |
| **F4** | Int.1 (F,16); Int.3 (M,15) | *Unmet mental health: 'They assume we have no problems' (Int.1); 'Once or twice a year does not accomplish anything' (Int.1); wants 'something meaningful' (Int.3)* | **B27** | Youth mental health gap, identified independently by both adolescents |
| **F4** | Int.2 (F,65); Int.6 (M,67) | *Service gaps: transport, specialists, elderly provision: 'There should be some provision for the elderly' (Int.2)* | **B26, B28, B29** | Multi-dimensional gaps for ageing populations |
| **F4** | Int.5 (F,52); Int.7 (M,48) | *Unmet psychological support: 'It would help to have a professional space, but it's not something we're used to' (Int.5)* | **B27** | Mid-life mental health, acknowledged but unaddressed |
| **F4** | Int.7 (M,48); Int.8 (M,37) | *Need for early mobilization: 'Many of us endure but are not well, not easily visible' (Int.7); 'It would help to be mobilized earlier' (Int.8)* | **B27, B28** | Demand for proactive health engagement, the PCNA's raison d'être |

***Note:*** *Int.1 = F, 16 yrs; Int.2 = F, 65 yrs; Int.3 = M, 15 yrs; Int.4 = F, 34 yrs (pregnant); Int.5 = F, 52 yrs; Int.6 = M, 67 yrs; Int.7 = M, 48 yrs; Int.8 = M, 37 yrs. Professional focus group findings (n = 31, 3 groups) are reported separately. Five cross-cutting themes emerged: (1) unexpressed needs (8/8 interviews), (2) demand for holistic PHC (7/8), (3) avoidance/postponement of prevention (6/8), (4) subthreshold psychological distress (8/8), (5) self-care deprioritization (6/8).*

**Table S3:** Item Reduction Pathway: From Qualitative Themes to Validated Instrument

| Stage | Method | N items | N factors | Items removed | Criteria |
|---|---|---|---|---|---|
| **1. Qualitative exploration** | Focus groups (IPA) + Interviews (thematic analysis) | Thematic domains identified (9 Objects of Concern + community themes) | — | — | Theoretical saturation; alignment with Andersen's BMHSU |
| **2. Item generation** | Theme-to-item translation + expert panel + cognitive interviews | 65 candidate items section B (sections C, D, E, F) | — | Life-stage sections C, D, E, F excluded from psychometric evaluation | Content validity; face validity; clarity; community engagement |
| **3. EFA (n = 520)** | Principal Axis Factoring, Varimax rotation | 33 items retained | 10 factors | 32 weak general items removed | Loadings < 0.40; cross-loadings ≥ 0.40; eigenvalue > 1 (Kaiser, 1960) |
| **4. CFA (n = 297)** | Maximum Likelihood, AMOS 18.0 | 29 items retained | 9 factors | 4 items removed: items 8, 20 (poor fit) + Factor 10 (2 items, $\alpha$ = 0.62) | Model fit: $\chi^2$/df = 1.675, RMSEA = .048, CFI = .92, TLI = .90, SRMR = .060 |

*Note: The life-stage-specific sections (C = Pregnancy, D = Adolescents, E = Parents of school-age children, F = Infant care) were developed based on qualitative findings but were not included in the psychometric evaluation. The decision to focus on the general section (B items) reflected the study's primary aim: to produce a unified, population-level assessment tool applicable across demographic groups. The general items demonstrated adequate psychometric properties and broad applicability. The life-stage-specific modules represent a promising avenue for future targeted validation and could serve as supplementary assessment components in subsequent research.*

**Appendix S1.** The final version of the Primary Care Needs Assessment (PCNA) Questionnaire

## Scoring and Administration Instructions

The PCNA questionnaire is designed to assess primary health care needs and to evaluate the services provided. Most items follow a Likert scale, where 1 corresponds to "Not at all" or "Very poor" and the maximum to "Very much" or "Very good." Items measuring frequency are scored on a scale from "Never" to "Daily." Open-ended items may be categorized as appropriate.

The questionnaire comprises nine factors: Expressed Needs (B.14–18), Enabling Factors (B.19–21), User Satisfaction (B.13, 22–24), Unmet Health Needs (B.26–29), Psychological Distress (B.3, 4, 6), Sexual Well-being (B.5, 7), Preventive Care Needs (B.8–10), Physical Health Status (B.1, 2, 25), and Contextual Constraints (B.11, 12).

For each factor, responses are analyzed separately, as the instrument is designed to assess needs profiles rather than to produce a single composite score. However, an overall needs distribution index may be derived if deemed appropriate by the researcher.

Negatively worded items requiring reverse scoring are those expressing negative perceptions or conditions. Specifically, items B11 ("To what extent does your financial situation negatively affect your health?") and B12 ("To what extent does your family situation negatively affect your health?") should be reverse-scored before analysis (i.e., on a 1–5 scale, 1 becomes 5, 2 becomes 4, etc.), so that higher values reflect more positive conditions.

Results may be used to inform improvements in Primary Health Care services. Data can be compared with prior studies to identify trends and changes, or to support health policy development.

# PRIMARY CARE NEEDS ASSESSMENT – PCNA

**Dear Sir/Madam,**

We would like to invite you to complete a questionnaire on your needs in Primary Health Care. This research is conducted within the framework of:

......................................................................................................................................

**What is the objective of this research?**
We invite you to share your views on health needs that, based on your experience, you consider to be priorities. Your views will be used to inform the development of empirical tools for assessing health needs. The aim is to generate evidence-based information and to assess the extent to which services provided by Primary Health Care units meet the actual needs of the population.

**Why am I being invited to take part?**
You are invited to participate because your experience is particularly valuable for our effort to better understand health needs. Our aim is to contribute to the effective use of existing Primary Health Care structures in relation to the actual needs of the population, while also contributing meaningfully to the everyday practice of health professionals.

**Do I have to take part?**
Participation is entirely voluntary. You may change your mind at any point during the research process and you may choose not to answer any question. You may withdraw from the study at any time without providing a reason. Your participation will not have any negative consequences, as no personal information will be disclosed.

**What will I need to do?**
If you decide to participate, you will be asked to spend approximately 5 minutes completing the questionnaire below.

**How will I benefit from the research?**
We believe that the information collected will contribute to a better assessment of health needs. By participating, you contribute to the meaningful investigation of an issue of everyday relevance that concerns us all.

**Are there any negative consequences or potential risks associated with my participation?**
Participation in this research is not expected to cause discomfort or expose participants to any risk and is comparable to participation in everyday activities.

**What will happen to the information I provide?**
The information collected will be anonymous and confidential and will be used exclusively for research purposes. It will not be possible to identify you from the data you provide through your responses. If you agree to participate, please note that your responses may be shared with other researchers beyond the research team, strictly for research purposes, while maintaining your anonymity. In addition, anonymized (non-identifiable) data collected during the research will be included in scientific studies and publications.

The electronic database containing the anonymized data you provide will be stored on the computers of the research team members, without access by other individuals.

In case you have any questions or concerns, please do not hesitate to contact us (contact person details):

Full Name: ________________________________

Telephone: ________________________________

Email: ___________________________________

We are at your disposal.

You may keep this information sheet for your records.

Thank you very much for your cooperation.

**The Research Team**

**PRIMARY CARE NEEDS ASSESSMENT (PCNA) QUESTIONNAIRE**

| | **A. The following questions concern demographic information. The data will be used exclusively for statistical analysis.** | |
|---|---|---|
| A1. | Gender | ❒ Male<br>❒ Female<br>❒ Other: [ ] |
| A2. | What year were you born? | |
| A3. | Nationality | |
| A4. | What is your marital status? | ❒ I live with my spouse/partner, without children at home<br>❒ I live with my spouse/partner, with children at home<br>❒ I live alone, without children at home<br>❒ I live alone, with children at home<br>❒ I live with my parents<br>❒ I live with a roommate<br>❒ Other: [ ] |
| A5. | Number of people living in the household | |
| A6. | Number of children | |
| A7. | What is the highest level of education you have completed? | ❒ Lower secondary education<br>❒ Upper secondary education<br>❒ Technical/vocational school<br>❒ Technological Educational Institute (TEI)<br>❒ University (AEI)<br>❒ Postgraduate studies<br>❒ Doctoral studies |
| A8. | What is your employment status? | ❒ Employed full-time<br>❒ Employed part-time<br>❒ Unemployed<br>❒ Student<br>❒ Retired<br>❒ Homemaker<br>❒ Self-employed<br>❒ Other: [ ] |
| A9. | What is your monthly household income? | ❒ Less than 1000<br>❒ 1001–2000<br>❒ 2001–3000<br>❒ 3001 or more<br>❒ Other: [ ] |
| A10. | What is your religion? | |

| | | |
|---|---|---|
| A11. | Do you have any chronic health condition? | ❒ No<br>❒ Disability<br>❒ Autoimmune disease<br>❒ Allergies<br>❒ Respiratory problems<br>❒ Diabetes<br>❒ Hypertension<br>❒ Heart disease<br>❒ Arthritis<br>❒ Other: [ ] |
| A12. | Do you have access to the internet? | ❒ Yes<br>❒ No |
| A13. | Are you registered with a public Primary Health Care service? | ❒ Yes<br>❒ No<br>❒ I don't know |

| | | | | |
|---|---|---|---|---|
| A14. | Do you seek care in the public sector, the private sector, or both for health issues? | ❒ Public | ❒ Private | ❒ Both public and private |
| A15. | Is your above choice based on: | ❒ Financial reasons | ❒ Quality of services | ❒ Both |
| A16. | Have you had an accident recently? | | ❒ Yes | ❒ No |
| A17. | Have you had any surgical procedures in the past? | | ❒ Yes | ❒ No |

**B. The following questions concern your physical and mental health over the past year. For each question, please select the response that best represents you.**

| Very poor<br>1 | Poor<br>2 | Fair<br>3 | Good<br>4 | Very good<br>5 |
|---|---|---|---|---|

| | | |
|---|---|---|
| B1. | How would you rate your overall physical health? | 1 2 3 4 5 |
| B2. | How would you rate your dietary habits in relation to your body's functioning? | 1 2 3 4 5 |
| B3. | How would you rate your psychological well-being? | 1 2 3 4 5 |
| B4. | How would you rate your management of stress in your daily life? | 1 2 3 4 5 |
| B5. | How would you rate your sexual life? | 1 2 3 4 5 |
| B6. | How would you rate your time management between work, family, and personal time? | 1 2 3 4 5 |

| Not at all<br>1 | A little<br>2 | Moderately<br>3 | Quite a lot<br>4 | Very much<br>5 |
|---|---|---|---|---|

| | | |
|---|---|---|
| B7. | Are you satisfied with your sexual life? | 1 2 3 4 5 |

| | | | | | | |
|---|---|---|---|---|---|---|
| B8. | How informed do you feel about sexually transmitted diseases and contraception? | 1 | 2 | 3 | 4 | 5 |
| B9. | Do you consider regular preventive screening to be useful? | 1 | 2 | 3 | 4 | 5 |
| B10. | How informed do you feel regarding preventive screening (e.g. breast self-examination, Pap test, prostate examination, etc.)? | 1 | 2 | 3 | 4 | 5 |
| B11. | To what extent does your family situation negatively affect your health? | 1 | 2 | 3 | 4 | 5 |
| B12. | To what extent does your financial situation negatively affect your health? | 1 | 2 | 3 | 4 | 5 |
| B13. | To what extent do the services provided by Primary Health Care structures meet your needs? | 1 | 2 | 3 | 4 | 5 |

| | | | | | | |
|---|---|---|---|---|---|---|
| For what reasons did you use Primary Health Care services over the past year? | B14. For routine medical check-ups and vaccinations | 1 | 2 | 3 | 4 | 5 |
| | B15. For the management of chronic conditions (such as diabetes, hypertension, heart disease, respiratory diseases, arthritis, etc.) | 1 | 2 | 3 | 4 | 5 |
| | B16. For prescription of medication | 1 | 2 | 3 | 4 | 5 |
| | B17. For diagnosis and specialized tests | 1 | 2 | 3 | 4 | 5 |
| | B18. For referral to medical specialists | 1 | 2 | 3 | 4 | 5 |
| | B19. Because I feel familiarity and safety | 1 | 2 | 3 | 4 | 5 |
| | B20. For free and public services | 1 | 2 | 3 | 4 | 5 |
| | B21. Due to ease of access | 1 | 2 | 3 | 4 | 5 |

| | | | | | | |
|---|---|---|---|---|---|---|
| B22. | Do you believe you have adequate access to health care when you need it? | 1 | 2 | 3 | 4 | 5 |
| B23. | Are you satisfied with the health care system in your community? | 1 | 2 | 3 | 4 | 5 |
| B24. | Is there an adequate number of health and social services in your community? | 1 | 2 | 3 | 4 | 5 |

| **Never** | **Rarely** | **A few times per month** | **A few times per week** | **Daily** |
|---|---|---|---|---|
| **1** | **2** | **3** | **4** | **5** |

| | | | | | | |
|---|---|---|---|---|---|---|
| B25. | How often do you engage in physical activity? | 1 | 2 | 3 | 4 | 5 |

| **Not at all** | **A little** | **Moderately** | **Quite a lot** | **Very much** |
|---|---|---|---|---|
| **1** | **2** | **3** | **4** | **5** |

| | | | | | | |
|---|---|---|---|---|---|---|
| In relation to your health and quality of life, which services do you believe are lacking in Primary Health Care in your area? | B26. Medical specialties | 1 | 2 | 3 | 4 | 5 |
| | B27. Mental health services | 1 | 2 | 3 | 4 | 5 |
| | B28. Health information / preventive screening | 1 | 2 | 3 | 4 | 5 |
| | B29. Transportation services to health care facilities | 1 | 2 | 3 | 4 | 5 |

**Thank you very much for your participation in this research!**
